# Morphological and Hemodynamic Risk Factors for the Rupture of Proximal Anterior Cerebral Artery Aneurysms (A1 Segment)

**DOI:** 10.3389/fnagi.2022.835373

**Published:** 2022-02-18

**Authors:** Mingwei Xu, Nan Lv, Kai Sun, Rujun Hong, Hao Wang, Xuhui Wang, Lunshan Xu, Lizhao Chen, Minhui Xu

**Affiliations:** ^1^Department of Neurosurgery, Daping Hospital, Army Medical University, Chongqing, China; ^2^Department of Neurosurgery, Changhai Hospital, Naval Medical University, Shanghai, China

**Keywords:** intracranial aneurysms, proximal anterior cerebral artery (A1 segment), morphology, hemodynamics, computational fluid dynamics

## Abstract

**Objective:**

The treatment of unruptured small intracranial aneurysms remains controversial. A distinguishing characteristic of A1 segment aneurysms is that they tend to rupture when they are small, which may be related to their distinctive morphology and hemodynamics. Our study sought to investigate the rupture risk factors of A1 segment aneurysms by analyzing the clinical risk factors, morphology, and hemodynamic characteristics of A1 segment aneurysms.

**Methods:**

We retrospectively enrolled 49 (23 ruptured, 26 unruptured) consecutive patients presenting to our institute with A1 segment aneurysms between January 2010 and March 2020. Independent risk factors associated with the rupture of A1 segment aneurysms were analyzed by multivariate regression analysis in the ruptured group and unruptured group.

**Results:**

Clinical risk factors, including age, sex, hypertension, smoking history, and SAH family history revealed no difference between the ruptured and unruptured groups. The ruptured group presented a significantly larger size (Size, *P* = 0.007), aspect ratio (AR, *P* = 0.002), size ratio (SR, *P* = 0.001), bottleneck index (BN, *P* = 0.016), dome-to-neck ratio (DN, *P* = 0.001), and oscillatory shear index (OSI) (*P* = 0.001) than the unruptured group. The normalized wall shear stress (NWSS) of the ruptured aneurysms was lower than the unruptured group (*P* = 0.001). In the multivariate regression analysis, only SR (OR = 3.672, *P* = 0.003) and NWSS (OR = 0.474, *P* = 0.01) were independent risk factors in the A1 segment aneurysm rupture.

**Conclusion:**

A higher SR and lower NWSS revealed a close connection with the rupture of A1 segment aneurysms in our study, thus providing a reference for clinical decision-making in treating A1 segment unruptured aneurysms.

## Introduction

The incidence of the aneurysms arising from a proximal anterior cerebral artery (A1 segment) is relatively low, with an incidence of 0.59–4%, as reported in the literature (Wakabayashi et al., 1985; Suzuki et al., 1992; Wanibuchi et al., 2001; Dashti et al., 2007; Lee et al., 2010; Park et al., 2013; Bhaisora et al., 2014; Maiti et al., 2016; Ding et al., 2017; Kim and Lim, 2019). A1 segment aneurysms are characterized by small size at rupture, multiplicity, associated vascular anomaly, and many incorporating perforators (Suzuki et al., 1992; Dashti et al., 2007; Lee et al., 2010; Park et al., 2013). Clinically, most ruptured A1 segment aneurysms are small, with a maximum size of < 5 mm, which stands in contrast with previous studies suggesting that small intracranial aneurysms (<5 mm) should be managed conservatively (Wiebers et al., 2003; Juvela et al., 2008; Investigators et al., 2012). Furthermore, A1 segment aneurysms are closely related to the perforating arteries around them, which may affect the perforating arteries and result in serious neurological dysfunction. Therefore, A1 segment aneurysms are dangerous, and it is significant to assess the risk factors of A1 segment aneurysm rupture. Aneurysm ruptures are reported to be related to morphological and hemodynamic parameters; however, results remain highly controversial (Dhar et al., 2008; Kashiwazaki and Kuroda, 2013; Duan et al., 2016; Lv et al., 2016b). This may be because most of these studies are not location-specific, and aneurysms at different locations may have distinctive morphological and hemodynamic characteristics. Due to their extremely rare occurrence, there are few published studies on the morphological and hemodynamic characteristics of A1 segment aneurysms. Our study focused on A1 segment aneurysms and investigated the potential risk factors for aneurysm rupture, including morphology and hemodynamics.

## Materials and Methods

### Patient Selection and Clinical Characteristics

We reviewed the clinical data of 2,247 aneurysms in 1,987 patients admitted to our institute between January 2010 and September 2020. The patient selection criteria were as follows:

Inclusion criteria: (1) Patients with A1 saccular aneurysms, (2) Complete clinical medical records; (3) Clinical or imaging evidence for the presence or absence of subarachnoid hemorrhage; (4) CTA image data meet the need for morphological and hemodynamic analysis.

Exclusion criteria: (1) Patients with A1 or fusiform aneurysms; (2) Patients with multiple aneurysms and the distance between aneurysms were too close to each other for CFD simulation, such as an anterior communicating aneurysm; (3) Patients with other cerebrovascular diseases, including arteriovenous malformation and arteriovenous fistula, etc.; (4) CTA data could not meet the needs of morphological and hemodynamic analysis.

Of the 1,987 patients, 52 had A1 segment aneurysms. Of the 52 A1 segment aneurysms, 1 case of blood blister aneurysms and 2 cases of fusiform aneurysms were excluded from the comparative analysis because it was difficult to study their morphological and hemodynamic indicators. Among the 49 A1 segment aneurysms, 23 were identified as ruptured aneurysms and 26 as unruptured aneurysms based on the evidence of subarachnoid hemorrhage (SAH) on a CT scan. This study assessed the currently recognized clinical risk factors for the rupture of aneurysms, including age, sex, hypertension, diabetes, smoking history, and family history of familial SAH. The definitions of hypertension, diabetes, and smoking history were by those laid forth by the previous articles of our research group (Lv et al., 2016a).

### Measurement and Calculation of Morphological Characteristics

**FIGURE 1:**
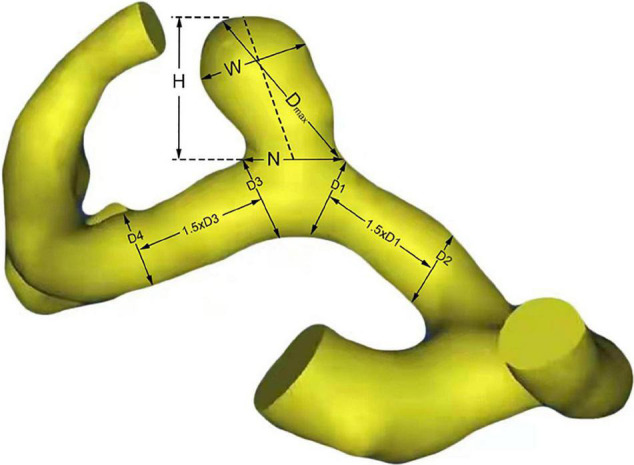
Definitions of morphological parameters. H, W, and N represent the height, width and neck size of aneurysm, respectively. Size = Dmax; Aspect ratio (AR) = H/N; Size ratio (SR) = 4Dmax/(D1 + D2 + D3 + D4); Dome-to-neck ratio (DN) = Dmax/N; height-to-width ratio (HW) = H/W; Bottleneck factor = Width/Neck.

There are six morphological parameters used in the study, including size, AR, SR, DN, height-to-width ratio (HW), bottleneck factor (BN), which were defined as previously reported (Hoh et al., 2007; Dhar et al., 2008). The size, height, width, and diameter of the artery of the aneurysm were measured, as shown in Figure 1. The maximum distance and the vertical distance from the aneurysm dome to the neck plane are defined as the size and the height of the aneurysm, respectively. And the width is the maximum diameter of the body perpendicular to the height. The DICOM data of patients’ CTA were analyzed by Mimics 17.0 (Mimics 17.0, Materialize, Belgium) to reconstruct 3D models of A1 segment aneurysms. Calculation of morphological parameters was performed using MATLAB 9.2 (MathWorks, Natick, Massachusetts, United States).

Other radiologic findings, including irregular shape, hypoplasia/aplasia of A1, fenestration of A1, and multiplicity, were also included in this study to investigate their relationship with the rupture of the A1 segment aneurysm. An irregular shape of an aneurysm was defined as a lobulated aneurysm or small bleb protruding from the aneurysm sac. Hypoplasia of A1 was defined as the diameter of the ipsilateral A1, which was less than 50% of the contralateral side. The irregular shape and vascular anomalies were assessed using a high-resolution CTA.

### Computational Fluid Dynamic Simulating and Hemodynamic Parameter Calculations

Geomagic Studio 9.0 (3D Systems, Inc.) and ICEM CFD 11.0 (ANSYS, Inc.) were used for the 3D models building of A1 aneurysms. As previously described, each vessel model was divided into the aneurysm, parent artery, and other vessels (Xu et al., 2013). The number of volume element grids for each 3D model was approximately between 1000,000 and 1400,000. Computational Fluid Dynamic (CFD) simulations were performed by CFX 11.0 (ANSYS, Inc.), assuming that the blood flow is laminar and incompressible and the vessel is rigid (Valencia et al., 2006). The density of the blood flow was 1,050 kg/m^3^ and the dynamic viscosity was 0.00345 Pa⋅s. According to a transcranial Doppler from a healthy subject, the flow inlet boundary conditions were set as typical pulsatile velocity profile (He and Ku, 1996) and the outlet boundary conditions were set as opening with zero static pressure (Lv et al., 2020). The total cardiac cycle was set to 0.8 s with a time step of 0.001 s, and the numeric simulation was performed by two consecutive cardiac cycles. The output was set as the result from the last cycle.

Quantitative hemodynamic parameters involved in this study include normalized wall shear stress (NWSS), the oscillatory shear index (OSI), the ratio of low WSS area (LSAR, below 10% of the mean WSS), normalized pressure (NP), and relative residence time (RRT) (Xiang et al., 2011).

### Statistical Analysis

SPSS version 23.0 (IBM Corp, Armonk, NY, United States) and Microsoft Excel 2013 were used for statistical analyses. The parameters were expressed as the number of patients (%) or median (interquartile range). The chi-square test and the Mann-Whitney *U*-test were performed for categorical variables and quantitative data, respectively. To assess independent risk factors for the rupture of A1 segment aneurysms, multivariate logistic regression analysis was performed for all relevant parameters. Statistical significance considered significant if *P* < 0.05.

## Results

### Clinical Characteristics

In this study, 49 patients with A1 segment aneurysms were included (16 men and 33 women) with a mean age of 60 years (range, 37–75 years). A total of 49 cases of A1 segment aneurysms were divided into the ruptured group (23 cases) and the unruptured group (26 cases) according to the evidence of SAH in the CT scan. The clinical characteristics of the 49 A1 segment aneurysms are listed in Table 1. No significant differences were detected in baseline characteristics (including age, sex, hypertension, diabetes, smoking history, and family history of SAH) between the two groups, thereby indicating that the morphological and hemodynamic characteristics were comparable.

**TABLE 1:** Clinical characteristics of A1 aneurysms.

	A1 segment aneurysms			
Variables	Total (*n* = 49)	Ruptured (*n* = 23)	Unruptured (*n* = 26)	*P*-value
**Clinical characteristics**				
Age (Years)	60 (51, 67)	57 (47, 64)	62 (57, 68)	0.078
Male, n (%)	16 (32.65%)	8 (34.78%)	8 (30.77%)	0.765
Hypertension, n (%)	26 (53.06%)	10 (43.48%)	16 (61.854%)	0.206
Diabetes, n (%)	5 (10.2%)	2 (8.69%)	3 (11.56%)	0.743
Smoking, n (%)	8 (16.33%)	4 (17.39%)	4 (15.38%)	0.85
Earlier SAH, n (%)	3 (6.12%)	1 (4.35%)	2 (7.69%)	0.929

### Morphological and Hemodynamic Parameters

The characteristics of morphology and hemodynamics of the 49 A1 segment aneurysms are represented in Table 2. In terms of morphological indices, significant differences were defined between the ruptured and unruptured groups. A larger size (*p* = 0.007), SR (*p* = 0.001), AR (*p* = 0.002), DN (*P* = 0.001), and BN (*P* = 0.016) and a significantly higher proportion of aneurysms with irregular shapes (*P* = 0.008) were found in the ruptured group. There were no significant differences in other morphological characteristics of vascular anomalies, including hypoplasia/aplasia of A1 (*P* = 0.5252), fenestration of A1 (*P* = 0.873), and multiplicity (*P* = 0.566) between the ruptured and unruptured groups.

**TABLE 2:** Morphological and hemodynamic characteristics of A1 aneurysms.

	A1 segment aneurysms			
Variables	Total (*n* = 49)	Ruptured (*n* = 23)	Unruptured (*n* = 26)	*P*-value
Morphological characteristics				
Size (mm)	3.07 (2.47, 3.85)	3.51 (2.78, 4.45)	2.69 (2.12, 3.45)	0.007
Aspect ratio (AR)	1.06 (0.83, 1.24)	1.21 (1.07, 1.35)	0.9 (0.72, 1.08)	0.002
Size ratio (SR)	1.41 (1.07, 1.81)	1.60 (1.38, 2.21)	1.12 (0.92, 1.53)	0.001
Dome-to-neck ratio (DN)	1.17 (0.83, 1.24)	1.36 (1.19, 1.64)	1.04 (0.84, 1.18)	0.001
Bottleneck index (BN)	1.10 (1.00, 1.25)	1.18 (1.05, 1.42)	1.04 (0.89, 1.17)	0.016
Height-width ratio (HW)	0.93 (0.80, 1.07)	0.98 (0.85, 1.13)	0.88 (0.84, 0.94)	0.161
Irregular shape	11 (22.45%)	9 (39.13%)	2 (7.69%)	0.008
Hypoplasia/aplasia of A1	17 (34.69%)	10 (43.48%)	9 (34.62%)	0.525
Fenestration of A1	6 (12.24)	3 (13.04%)	3 (11.54%)	0.873
Multiplicity	9 (18.37%)	5 (21.74%)	4 (13.79%)	0.566
Hemodynamic characteristics				
Normalized WSS	0.68 (0.44, 0.84)	0.57 (0.53, 0.66)	0.73 (0.64, 0.89)	0.001
Percentage of low WSS area (LSA)	0 (0.00, 0.004)	0.0001 (0.00, 0.046)	0 (0.00, 0.0016)	0.088
Oscillatory shear index	0.0116 (0.0073, 0.0198)	0.0192 (0.0112, 0.0269)	0.0084 (0.0058, 0.0136)	0.001
Pressure	1.18 (1.06, 1.30)	1.21 (1.06, 1.31)	1.16 (1.06, 1.31)	0.869
Relative residence time	0.20 (0.13, 0.42)	0.27 (0.15, 0.50)	0.18 (0.11, 0.23)	0.175

*Variables are expressed as median (interquartile range), or number of patients (%).*

**FIGURE 2:**
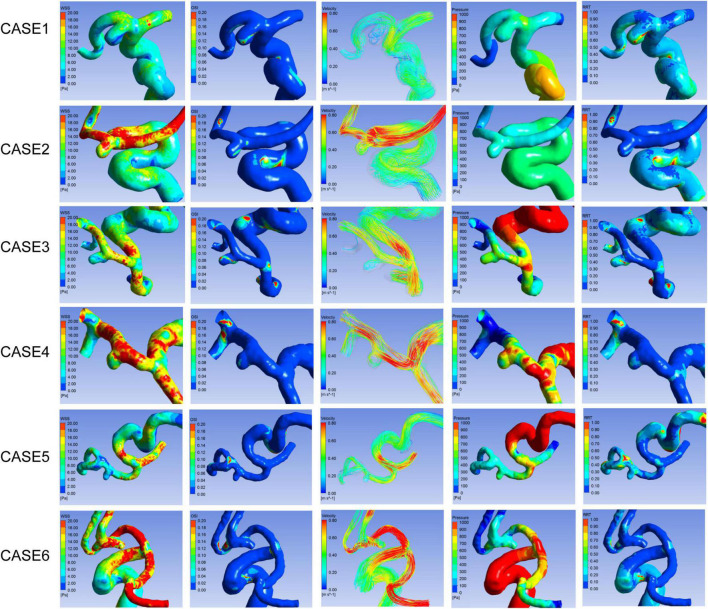
Hemodynamic patterns of six representative A1 segment aneurysms. Cases1, 3, and 5 were ruptured aneurysms, and 2, 4, and 6 were unruptured aneurysms. Cases 1 and 2, 3 and 4, 5 and 6 located in proximal, middle, and distal segment of A1, respectively. From left to right shows distribution of WSS, OSI, Pressure, RRT, and flow pattern at the systolic peak of each aneurysm respectively.

In the comparative analyses of hemodynamic parameters, the ruptured A1 segment aneurysms were proven to have a significantly lower normalized WSS (*P* = 0.001) and higher OSI (*P* = 0.001) than the unruptured group. Other hemodynamic characteristics, including the percentage of low WSS area (LSA) (*P* = 0.088), pressure (*P* = 0.869), and RRT (*P* = 0.175), revealed no significant differences. Hemodynamic patterns of six representative cases are shown in Figure 2.

### Multivariate Analysis

To identify independent risk factors of A1 segment aneurysm rupture, all differential morphological and hemodynamic indicators were included in a further multivariate regression analysis. Finally, our study revealed that independent variables of the rupture status of A1 segment aneurysms were SR (odds ratio = 3.672; *p* = 0.003) and normalized WSS (odds ratio = 0.474; *p* = 0.01) in Table 3.

**TABLE 3:** Independent risk factors for rupture of A1 aneurysms.

Variables	*P*-value	Odds ratio	95% confidence interval
Size ratio (SR)	0.003	3.672	1.578–8.540
Normalized WSS	0.010	0.0474	0.268–0.837

## Discussion

The incidence of A1 segment aneurysms is relatively low, accounting for about 0.59–4% of all intracranial aneurysms (Wakabayashi et al., 1985; Suzuki et al., 1992; Wanibuchi et al., 2001; Dashti et al., 2007; Maiti et al., 2016; Ding et al., 2017). In this study, A1 segment aneurysms accounted for 2.61% of all aneurysms and 2.31% of all aneurysms diagnosed during the study period. However, owing to the extremely rare occurrence, little attention has been paid to A1 aneurysms, especially to their risk factors for rupture. To our knowledge, it is the first study to identify possible risk factors for A1 segment aneurysm rupture by combining morphological and hemodynamic analyses.

The majority of small unruptured intracranial aneurysms (IAs) are increasingly being detected due to advanced imaging techniques. The International Study of Unruptured Intracranial Aneurysms (ISUIA) report elucidates that the annual rupture risk of UIAs ≤ 6 mm is very low (0.069%), while the rupture risk of UIAs > 7 mm increases to 2.6% (Wiebers et al., 2003). Previous studies have suggested that a high rupture rate of A1 segment aneurysms, which are prone to rupture at smaller sizes (Suzuki et al., 1992; Dashti et al., 2007; Lee et al., 2010; Park et al., 2013). In the study by Kim and Lim (2019), the average size of 30 A1 aneurysms was 4.1 mm; however, the rupture rate was as high as 40%. In Maiti et al. (2016) reported a series of 16 consecutive A1 segment aneurysms with a rupture rate as high as 50%, but the average rupture size of the rupture group was only 4.38 mm. In this series here, the greatest size of a ruptured A1 aneurysm was 6.59 mm, while the median size was 3.51 mm, and the rupture rate was as high as 46.94%. Moreover, A1 aneurysms are usually involved or even attached to many small perforators, which can incur serious neurologic deficits once they rupture. These findings indicate that A1 segment aneurysms are dangerous even if they are small; however, clinical treatment of unruptured small A1 aneurysms remains controversial for physicians, and reliable predictors are required to assess rupture risk.

Many studies have been carried out on the relationship between the risk of IAs rupture and morphology and hemodynamics, but the conclusions remain controversial, which may be related to the fact that the risk of IAs rupture varies across different locations (Forget et al., 2001). As a result, prior rupture risk factors may not necessarily apply to A1 segment aneurysms. Moreover, due to their rare incidence, previous studies on the rupture risk factors of A1 segment aneurysms have been limited. Kim and Lim (2019) pointed out that A1 segment aneurysms with irregular shapes, high aspect, or height-width ratio value showed a high risk of rupture by studying 32 cases of A1 aneurysms; however, hemodynamic variables were not included in this study. Xiang et al. (2011) found that the combination of morphological model (SR) and hemodynamics (WSS and OSI) could discriminate IA rupture status with high AUC values in a study of 119 IAs. Therefore, in this study, we only considered A1 aneurysms. Clinical risk factors, morphological indices, and hemodynamic indices of A1 aneurysms were studied together.

Previous studies have confirmed that clinical characteristics are closely related to the risk of IAs rupture (Bor et al., 2015). Our study demonstrated that clinical characteristics of age, sex, and hypertension showed no significant difference between the two groups, suggesting that the morphological and hemodynamic results were comparable. Another anatomical characteristic of A1 segment aneurysms is associated with vascular anatomical variations, including hypoplasia, aplasia, or fenestration of the A1 segment of the ACA, which may contribute to higher rupture rate of A1 aneurysms than arising from other location. Variant anatomies were reported in 21, 31.3, and 42.9% of patients with A1 segment aneurysms, in the studies by Suzuki et al. (1992); Bhaisora et al. (2014), and Maiti et al. (2016), respectively. In this study, 34.69% of the patients with A1 segment aneurysms had contralateral A1 hypoplasia or aplasia, and 12.24% of the patients were associated with fenestration of the A1 trunk, which is consistent with previous reports. However, the proportion of association of variant anatomy was not significantly different in this study. These results suggest that the variant anatomy of the A1 trunk may be related to the formation, but not the rupture of A1 segment aneurysms.

Previous studies have demonstrated that some morphological parameters are related to the rupture of intracranial aneurysms, among which aneurysm size is the most commonly used parameter in the clinical decision-making of aneurysm treatment. In our series of cases, the size of aneurysms was not statistically significant in the regression analysis, although it was larger in the ruptured group. Although AR, SR, DN, BN, and irregular morphology were statistically different in the univariate analysis, only SR was statistically different in the multivariate regression analysis, suggesting that a higher SR value is an independent risk factor for the A1 segment aneurysm rupture. This conclusion is consistent with previous results. In 2008, Dhar et al. (2008) proposed SR as a new indicator for predicting the rupture of intracranial aneurysms. Due to the inclusion of the morphology of aneurysmal parent arteries, rupture of intracranial aneurysms can be more accurately predicted than aneurysm size. Kashiwazaki and Kuroda (2013) also pointed out that for small aneurysms (<5 mm), SR values, rather than aneurysm size, are a better predictor of rupture risk. Rahman et al. (2010) also demonstrated in a prospective study that SR was associated with aneurysm rupture. Only A1 segment aneurysms were included in this study, most of which were small aneurysms. Our results also confirmed SR as an independent risk factor for rupture of A1 aneurysms.

Hemodynamics is thought to be closely related to the occurrence, growth, and rupture of intracranial aneurysms (Duan et al., 2016). Previous studies have shown that low WSS and high OSI can promote endothelial surface adhesion molecules upregulation, inflammatory cell infiltration, and lead to tumor wall degradation and eventual rupture (Malek et al., 1999). Can and Du (2016) reported that high OSI was associated with the growth of the aneurysm. In this study, accepted hemodynamic indicators related to aneurysm rupture were also included, including NWSS, LSA, OSI, NP, and RRT. And our study demonstrated lower NWSS and higher OSI in the ruptured group. However, in the multi-factor regression analysis, only NWSS remained an independent risk factor for A1 segment aneurysm rupture. Previous studies on the relationship between hemodynamics and intracranial aneurysm rupture have been controversial, which may be due to the fact that these studies were not site-specific. At the same time, it has been pointed out that hemodynamic parameters combined with morphological parameters could predict the IAs rupture more accurately. Tremmel et al. (2009) reported that aneurysm hemodynamics with a higher SR are characterized by multiple vortices, more complex flow patterns, and lower NWSS in the apex area, which may be associated with aneurysm rupture. In this study, only A1 segment aneurysms were included, and morphological and hemodynamic indicators were also included. The results confirmed that higher SR and lower NWSS were related to the A1 segment aneurysm rupture, which was consistent with the conclusion of Tremmel et al. (2009) and may provide a reference for clinical treatment of A1 segment unruptured aneurysms. This study demonstrated higher SR and lower NWSS values as independent risk factors for the A1 segment aneurysm rupture. However, several limitations existed in this study. Firstly, due to the extremely rare occurrence of A1 segment aneurysms, the relatively small sample size might have affected analyses. Morphological and hemodynamic parameters were used together for the reliability of the multivariate regression analysis. Secondly, as a retrospective study, the boundary conditions in hemodynamics analysis were not patient-specific, whereas the CFD simulation models were patient-specific.

## Conclusion

In this study, the characteristics of unruptured A1 segment aneurysms with ruptured A1 segment aneurysms were compared and higher SR and lower NWSS values were identified as discriminators of the rupture status of A1 aneurysms. This may provide a reference for clinical treatment of A1 segment unruptured aneurysms.

## Data Availability Statement

The original contributions presented in the study are included in the article/supplementary material, further inquiries can be directed to the corresponding author/s.

## Ethics Statement

The studies involving human participants were reviewed and approved by the Ethics Committee of Army Specialty Medical Center, Army Medical University. The patients/participants provided their written informed consent to participate in this study. Written informed consent was obtained from the individual(s) for the publication of any potentially identifiable images or data included in this article.

## Author Contributions

MingX, KS, and RH collected the patients’ clinical data. MingX and NL performed morphological analysis and computational fluid dynamics simulation. XW and HW completed the data statistics. MingX and RH drafted the manuscript. LC and LX designed the research project and revised the manuscript with MinhX. MinhX and HW participated in the case diagnosis. All authors read and approved the final manuscript.

## Conflict of Interest

The authors declare that the research was conducted in the absence of any commercial or financial relationships that could be construed as a potential conflict of interest.

## Publisher’s Note

All claims expressed in this article are solely those of the authors and do not necessarily represent those of their affiliated organizations, or those of the publisher, the editors and the reviewers. Any product that may be evaluated in this article, or claim that may be made by its manufacturer, is not guaranteed or endorsed by the publisher.
